# Isolation, Identification and Measurement of Virulence Against the Potato Tuber Moth, *Phthorimaea operculella*, of *Metarhizium robertsii* ML-2

**DOI:** 10.3390/insects17050474

**Published:** 2026-05-04

**Authors:** Lingying Zhang, Zaotang Su, Yaning Wang, Zhao Qiu, Yijie Zuo, Yuhe Dai, Bin Chen, Guanli Xiao

**Affiliations:** 1 College of Plant Protection, Yunnan Agricultural University, Kunming 650201, China; 15969467250@163.com (L.Z.); 18468181679@163.com (Z.S.); 13354943980@163.com (Y.D.); 2 College of Agronomy and Life Sciences, Zhaotong University, Zhaotong 657000, China; 3 College of Agronomy and Biotechnology, Yunnan Agricultural University, Kunming 650201, China; 15946786115@163.com (Y.W.); 18487431840@163.com (Z.Q.); 15094209525@163.com (Y.Z.)

**Keywords:** *Phthorimaea operculella*, *Metarhizium robertsii*, molecular identification, Time-Concentration-Mortality Model, biological control

## Abstract

*Phthorimaea operculella* is a globally distributed pest of solanaceous crops and represents one of the most critical constraints on potato production worldwide. The overuse of chemical insecticides has led to widespread resistance in pest populations, making entomopathogenic fungi a promising alternative strategy for sustainable agricultural pest management. In this study, a fungal strain designated ML-2 was isolated from naturally infected *P. operculella* larvae collected from a potato field in Yunnan province, China. Through morphological characteristics and ITS sequence analysis, strain ML-2 was identified as *M. robertsii*. The growth rate and sporulation yield on PDA and SDAY media were assessed, the virulence of ML-2 against eggs, larvae, and pupae of *P. operculella* were determined using the dipping method, and the data were analyzed via the time-concentration-mortality (TCM) model. The results showed that there were no significant differences in the growth rate of strain ML-2 on the two culture media (*p* > 0.05), whereas the sporulation yield on PDA medium was significantly higher than that on SDAY medium (*p* < 0.01). The highest corrected cumulative mortality rates for *P. operculella* larvae and pupae were 94.50% and 83.07% on day 7 after inoculation, respectively, while the highest cumulative mortality rate for eggs was relatively low at only 20.28%. Additionally, at a concentration of 1 × 10^8^ spores/mL, the LT_50_ values of strain ML-2 against *P. operculella* larvae and pupae were 2.34 days and 2.72 days, respectively. On day 7 post-inoculation, the LC_50_ values for larvae and pupae were 3.24 × 10^7^ conidia/mL and 9.64 × 10^5^ conidia/mL, respectively. In conclusion, *M. robertsii* ML-2 exhibits high potential as a candidate biopesticide and can be used in the sustainable integrated management of *P. operculella*.

## Simple Summary

This study reports the natural infection of the agricultural pest Potato tuber moth, *Phthorimaea operculella*, by a *Metarhizium* species. Based on morphological identification and ITS molecular sequence analysis, the strain was identified as *Metarhizium robertsii* (Hypocreales: Clavicipitaceae) and designated as *Metarhizium robertsii* ML-2. Bioassays using the dipping method demonstrated that *M. robertsii* strain ML-2 exhibited strong virulence against the larvae and pupae of *P. operculella*. The conidial production of this strain on PDA medium was significantly higher than that on SDAY medium. These results indicate that *M. robertsii* strain ML-2 has considerable potential as an effective biocontrol agent against the larval and pupal of *P. operculella*.

## 1. Introduction

*Phthorimaea operculella* (Lepidoptera: Gelechiidae) is a globally distributed oligophagous pest, primarily infesting solanaceous crops such as potato and tobacco [1,2]. It is now distributed across multiple provinces in China, with particularly severe infestations frequently occurring in Yunnan, Guizhou, Sichuan, and other areas [3]. The larvae bore into and feed on potato roots, stems, and leaves. During storage, larval feeding creates numerous tunnels in potato tubers, severely reducing their quality and marketability, making this pest one of the most significant constraints on global potato production [4]. Excessive chemical insecticides application by growers, combined with continuous monoculture practices and the high reproductive capacity of its strong tendency to develop insecticide resistance, have rendered *P. operculella* difficult to control in both field cultivation and storage periods [5]. Therefore, the exploration of efficient and environmentally friendly biocontrol agents against this pest is of great practical significance and urgency.

Entomopathogenic fungi play a crucial role in regulating insect populations in natural ecosystems. Owing to their high insecticidal efficacy and environmental safety, these fungi are considered promising alternatives to chemical pesticides [6,7]. Among entomopathogenic fungi, members of the genus *Metarhizium* are regarded as highly promising due to their epizootic potential, broad host range, and environmental safety, playing a significant role in the development of green insecticides [8]. *Metarhizium* is a filamentous fungus that grows vegetatively as mycelia and infects hosts via conidia. Although the overall host range of *Metarhizium* is broad, considerable variation exists in the virulence of different strains against specific hosts. For example, an evaluation of 10 strains of *Metarhizium* anisopliae isolated from Australian soil against *Cylas formicarius* confirmed that, despite all strains belonging to the same species, only two strains caused over 50% mortality at 5 days post-inoculation, indicating that strain screening is a critical prerequisite for developing effective fungal insecticides [9]. An assessment of 30 *M. anisopliae* strains from Mexico revealed significant differences in growth rate, sporulation capacity, and virulence against *Galleria mellonella*, with some strains exhibiting high virulence, and most strains were capable of associating with plant roots [10]. Similarly, the virulence of 31 *Metarhizium rileyi* strains isolated from Yunnan Province against *Spodoptera frugiperda* varied significantly, with only a subset of strains showing high virulence. Genetic diversity analysis indicated significant genetic differentiation among strains, but no correlation between genetic distance and geographic distance, suggesting that virulence differences are associated with the individual genetic characteristics of the strains [11].

Furthermore, the virulence of a specific strain can also vary significantly depending on the host. For instance, a study comparing the virulence of the same *Metarhizium robertsii* strain against four different insect species, including *Tenebrio molitor*, *Locusta migratoria*, *Plutella xylostella*, and *Galleria mellonella*, demonstrated that the enhancement of virulence by the same strain varied significantly among different hosts [12]. Using *Metarhizium rileyi* strain Nr4 as a model, a study comparing its virulence against the larvae and pupae of *Spodoptera litura* confirmed that the same strain exhibits significant differences in virulence against different developmental stages of the same host [13]. Another study comparing the virulence of conidia from the same *M. anisopliae* strain against *T. molitor*, *Spodoptera frugiperda*, *Gryllus assimilis*, and *Apis mellifera* found that the strain exhibited the highest virulence against *T. molitor* and lower virulence against the other three insects, indicating that the pathogenicity of the same strain is highly selective among different hosts [14]. However, research on the insecticidal activity, growth characteristics, and sporulation capacity of specific *Metarhizium* strains against *P. operculella* remains limited. Therefore, in this study, a *Metarhizium* strain was isolated from naturally infected *P. operculella* larvae. Following identification, its virulence against the target pest and relevant biological characteristics were evaluated, aiming to provide a scientific basis for the further development and application of this strain.

In this study, a highly pathogenic fungal strain, designated ML-2, was isolated from naturally infected *P. operculella* larvae collected in the field. To further explore its biocontrol potential and expand its application scope, this study evaluated the efficacy of strain ML-2 against *P. operculella* eggs, larvae, and pupae through bioassays. The Time-Dose-Mortality (TDM) model was employed to analyze the time and concentration effects on larval and pupal mortality. Observations of disease symptoms in the insect hosts were also conducted. The aim was to identify the most susceptible developmental stage(s) for control, elucidate the temporal and concentration-dependent effects, and thereby assess the potential of *Metarhizium* strain ML-2 for controlling *P. operculella*.

## 2. Materials and Methods

### 2.1. Materials

Diseased insect specimens: Larvae of *Phthorimaea operculella* naturally infected and killed by entomopathogenic fungi were collected from a potato cultivation base in Malong District, Yunnan Province, China (25°18′42″ N, 103°44′6″ E; altitude: 2194 m). The specimens were transported to the Entomopathogen Laboratory, Yunnan Agricultural University, for further processing.

Sabouraud dextrose agar yeast extract (SDAY) medium: composed of 40 g of glucose, 10 g of peptone, 10 g of yeast extract powder, 20 g of agar, and 1000 mL of distilled (super-pure water) water. Potato dextrose agar (PDA) medium: prepared with 200 g potato (boiled and filtered), 20 g of glucose, 20 g of agar, and 1000 mL of distilled water.

Test Insect Source: *P. operculella* were collected from a potato cultivation base in Malong District, Yunnan Province, China. A laboratory colony was subsequently established on tubers of the potato variety ‘Cooperation 88’. For pupal collection, egg masses deposited within 24 h were placed in rearing cages supplied with fresh tubers of ‘Cooperation 88’. Following completion of larval development and pupation, uniform one-day-old pupae of similar size and vitality were selected. To obtain larval specimens, another set of egg masses was allowed to hatch, and the neonates were reared on leaves of ‘Cooperation 88’ until the third instar stage. Three-day-old larvae of consistent size and health were chosen for subsequent experiments. All insect colonies were maintained in climate-controlled chambers at 29 ± 1 °C under a 16 h light/8 h dark photoperiod.

### 2.2. Isolation of the Fungal Strain

Diseased larvae of *P. operculella* were placed in sterile Petri dishes (90 mm diameter) and maintained under humid conditions in a constant-temperature incubator at 25 °C. Following complete coverage of larval cadavers with green conidial masses, a small amount of conidia was collected using a sterile inoculation loop and streaked onto SDAY medium. The inoculated plates were incubated at 25 °C, 75% relative humidity, under a 16 h light/8 h dark photoperiod. Upon emergence of hyphae or conidia, a small quantity of conidia was transferred to fresh SDAY medium for purification. This procedure was repeated until colonies with uniform morphological characteristics were obtained. The purified isolate was designated as strain ML-2 and maintained on SDAY slant medium at 4 °C for subsequent experiments.

### 2.3. Morphological Characterization of Strain ML-2

Mycelial fragments of strain ML-2 were collected using a sterile syringe needle under aseptic conditions and inoculated onto SDAY and PDA media. Cultures were incubated at 25 °C and 75% relative humidity, under a 16 h light/8 h dark photoperiod. Upon emergence of mycelia and initial conidial formation on the medium surface, small mycelial samples were excised and fixed in 95% ethanol for 5 s, and then stained with Melzer’s reagent. Morphological features, including conidial shape, conidiogenous structures, and hyphal characteristics, were examined and photographed using a Leica light microscope (DM750 hardware; LAS 4.8 software), following the taxonomic guidelines described in Insect Mycology [15]. The observed morphological traits were compared with those reported for *Metarhizium robertsii* by Bischoff [16].

### 2.4. Methods for Molecular Identification of Strain ML-2

Genomic DNA of strain ML-2 was extracted using the cetyltrimethylammonium bromide (CTAB) method [17]. The internal transcribed spacer ITS region of the ribosomal DNA was amplified with the universal fungal primers ITS1 and ITS4. Meanwhile, the EF1-α region was amplified using the universal primers EF1α-EF and EF1α-ER (Table S1). PCR amplification was performed in a 25.0 µL reaction volume containing 12.5 µL of 2× Taq PCR Master Mix, 1.0 µL of each primer (10 µM), 1.0 µL of DNA template, and 9.5 µL of ddH_2_O. The thermal cycling conditions consisted of an initial denaturation at 95 °C for 3 min, followed by 35 cycles of denaturation at 94 °C for 1 min, annealing at 55 °C for 1 min, and extension at 72 °C for 1.5 min, with a final extension at 72 °C for 10 min. PCR products were examined by electrophoresis on 1.0% agarose gels, and amplicons of expected size were purified and subjected to bidirectional sequencing at Sangon Biotech Co., Ltd. (Shanghai, China). 

### 2.5. Phylogenetic Analysis

The raw sequences were assembled and manually edited using ContigExpress software to correct ambiguous base calls and remove terminal regions of poor quality. The resulting consensus sequence was compared against the NCBI database using BLAST (https://blast.ncbi.nlm.nih.gov/Blast.cgi/ accessed on 21 April 2026). The gene sequences amplified in this study were deposited in GenBank to obtain accession numbers. *M. robertsii* belongs to the PARB clade, a well-defined group of closely related entomopathogenic fungi. Therefore, for multi-gene phylogenetic analysis, ITS and EF1-α sequences of type or reference strains of PARB species were retrieved from the NCBI database as references (Table S2). The obtained sequence datasets were aligned, gaps were coded as “N”, and the aligned gene matrices were concatenated. Phylogenetic reconstruction was performed using the maximum likelihood (ML) method in MEGA X software, with *Torrubiella luteorostrata* NHJ12516 as the outgroup. Nodal support was assessed via bootstrap analysis with 1000 pseudoreplicates [18].

### 2.6. Mycelial Growth and Conidial Yield of Strain ML-2 on Different Culture Media

Conidia of strain ML-2 were harvested by scraping the colony surface with a sterile scalpel and suspended in 0.05% Tween-80 solution. The suspension was filtered to remove mycelial debris, and the conidial concentration was determined using a hemocytometer (XB-K-25). The concentration was adjusted to 1 × 10^7^ conidia/mL. An aliquot of 0.01 mL of the conidial suspension was inoculated onto PDA and SDAY media. Each medium treatment was replicated five times. All cultures were incubated at 25 °C and 75% relative humidity, under a 16 h light/8 h dark photoperiod. Colony diameter was measured every three days using the cross method over a 15-day period.

On day 15, the entire colony was scraped from the medium surface using a sterile scalpel and transferred into a conical flask containing 25 mL of sterile 0.05% Tween-80 solution. The suspension was homogenized and then subjected to ultrasonic oscillation for 3 min. Mycelial fragments were removed by filtration through sterile gauze, and the conidial concentration in the filtrate was quantified using a hemocytometer (XB-K-25). The conidial yield per plate was calculated accordingly.

### 2.7. Methods for Determining Virulence of Strain ML-2 Against Different Life Stages of P. operculella

Conidia of strain ML-2 were harvested by scraping the colony surface with a sterile scalpel, which were then suspended in 0.05% Tween-80 solution. The suspension was filtered to remove mycelial fragments and conidial concentrations were determined using a hemocytometer (XB-K-25). Meanwhile, 100 µL of the spore suspension was spread onto PDA plates and incubated at 25 °C for 24 h. Spores were considered germinated when the germ tube length exceeded half the spore diameter, and only suspensions with a viability greater than 90% were deemed suitable for virulence testing. Serial dilutions were prepared to obtain final concentrations of 1 × 10^8^, 1 × 10^7^, 1 × 10^6^, 1 × 10^5^, 1 × 10^4^, and 1 × 10^3^ conidia/mL. Virulence assays against eggs, larvae, and pupae of *P. operculella* were conducted following specific protocols, with 0.05% Tween-80 alone serving as the control treatment for all three bioassays. All treatments and controls were incubated under a 16 h light/8 h dark photoperiod, and mortality was monitored daily for 7 days.

For egg bioassays, 1-day-old eggs were immersed in the conidial suspensions for 10 s, then transferred to sterile Petri dishes and maintained under humid conditions. Under healthy conditions, the entire egg stage of *P. operculella* lasts only 4 days. Eggs that did not hatch within 7 days post-treatment and showed mycelial growth on the surface were recorded as dead. Each replicate consisted of 25 eggs, and the experiment was repeated three times [19,20]. For larval bioassays, uniform 3-day-old larvae of consistent size and vigor were immersed in the conidial suspensions for 10 s, then reared on fresh potato leaves. Larvae showing no movement within 5 s after careful probing with a fine brush were scored as dead. Cadavers were placed under humid conditions to confirm fungal outgrowth. Each replicate included 25 larvae, and the experiment was repeated three times [21]. For pupal bioassays, 1-day-old pupae of uniform size and health were immersed in the conidial suspensions for 10 s, then placed in cell culture plates lined with sterile moistened cotton. Pupae showing no response within 5 s after careful stimulation with forceps tips were recorded as dead, and dead pupae were maintained under humid conditions to observe sporulation. Each replicate comprised 25 pupae, and the experiment was repeated three times [22]. Mortality and corrected mortality were calculated.

$$Mortality\;rate\;(\%)=\frac{Number\;of\;dead\;insects}{Number\;of\;test\;insects}\times 100$$

$$Corrected\;mortality\;rate\;(\%)=\frac{Treatment\;group-control\;group}{1-control\;group}\times 100$$

### 2.8. Data Analysis

Experimental data were analyzed using SPSS 20.0 and Microsoft Excel 2016. Mortality rates were first subjected to one-way analysis of variance (ANOVA) to test for overall differences among treatment groups. When significant differences were detected (*p* < 0.05), Tukey’s honestly significant difference (HSD) test was used for multiple comparisons between groups. Image processing was carried out with Adobe Photoshop 2021, and figure composition and layout were completed using Adobe Illustrator CC 2018.

To analyze the responses of *P. operculella* larvae and pupae to different concentrations of *M. robertsii*, the time–concentration–mortality (TCM) model was employed to evaluate the interaction between strain ML-2 and the target insect stages. All modeling and computational procedures were conducted using DPS 14.0 software. The goodness-of-fit of the model was assessed using the Hosmer–Lemeshow statistic C, with a *p*-value ≥ 0.05 indicating that the fitted model passed the heterogeneity test and was considered an appropriate TCM model [22]. In this study, the model was fitted based on corrected mortality values, and the raw mortality data were corrected using the corrected mortality to eliminate the influence of natural mortality in the control group.

## 3. Results

### 3.1. Morphological and Molecular Identification

#### 3.1.1. Morphological Characteristics of Strain ML-2

**Figure 1:**
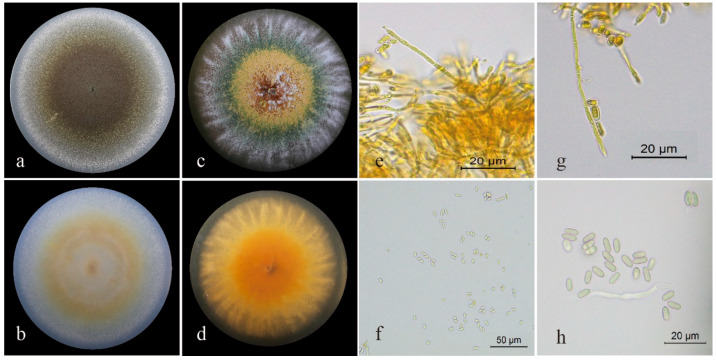
Morphological characteristics of strain ML-2. (**a**,**b**) Top-bottom face characteristics of strain ML-2 after 15 days of cultivation on PDA medium. (**c**,**d**) Top-bottom face characteristics of strain ML-2 after 15 days of cultivation on SDAY medium. (**e**,**g**) Mycelia and sporulation structures on SDAY medium at 40× magnification. (**f**) Conidia on SDAY medium at 40× magnification. (**h**) Conidia on SDAY medium at 100× magnification.

After 15 days of incubation on PDA medium, colonies of ML-2 were flat and densely powdery, with black centers and smooth white margins; the reverse sides were yellowish to white (Figure 1a,b). On SDAY, colonies showed concentric differentiation: the inner zone appeared yellow and felt-like, the middle zone green and powdery, and the outer zone white and velvety. The overall surface bore green powdery conidia overlaid with yellow felt-like hyphae; margins were broom-shaped and the reverse was yellow (Figure 1c,d). Hyphae were septate. Conidiophores arose vertically from substrate hyphae and produced conidia at their tips (Figure 1e,g). Conidia were elliptical, smooth-walled, and measured (6.17–7.64) × (2.33–3.14) μm (Figure 1f,h). No morphological differences in conidia were observed between the two culture media. Accordingly, only the sporulation structures, mycelia, and conidial morphology on SDAY medium are presented in Figure 1.

#### 3.1.2. Molecular Identification Results of Strain ML-2

**Figure 2:**
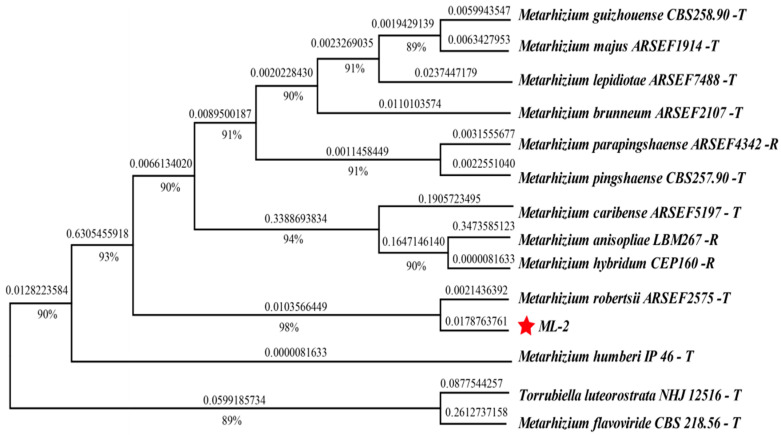
Phylogenetic tree based on ITS and EF1-α sequences, constructed using MEGA X with the maximum likelihood (ML) method. *Torrubiella luteorostrata* NHJ12516 was used as the outgroup. Bootstrap support values (1000 replicates) are shown as percentages at nodes, and branch lengths represent genetic distances (Kimura two-parameter model). “-T” and “-R” indicate type strain and reference strain, respectively. The red star indicates the strain isolated from diseased *Phthorimaea operculella* in this study.

ITS sequencing revealed that strain ML-2 exhibited 99.42% sequence identity and 97% query coverage with *Metarhizium robertsii* strain ARSEF2575 (NR132011), while EF1-α sequencing showed 98.87% identity and 99% coverage with the same strain (KX342729) (Table S3). Phylogenetic analysis based on the internal transcribed spacer (ITS) and elongation factor 1-alpha (EF1-α) regions demonstrated that strain ML-2 first clustered with *M. robertsii* ARSEF2575 on a distinct branch, clearly separated from other PARB species, with a bootstrap support value of 98% (Figure 2). These results indicate that strain ML-2 is more closely related to *M. robertsii*.

### 3.2. Effects of Different Media on Growth Rate and Sporulation of Strain ML-2

**Figure 3:**
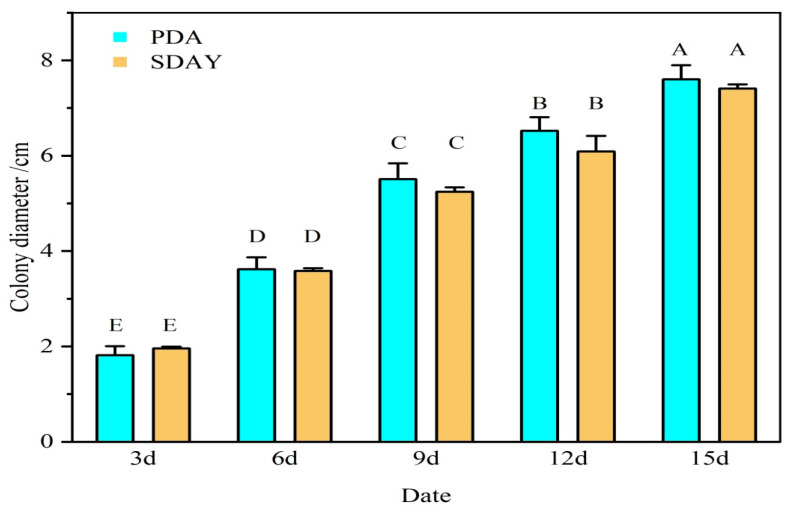
Growth rate of strain ML-2 on different media. Different uppercase letters or lowercase letters indicate a significant difference at the 0.01 level or a significant difference at the 0.05 level (Duncan’s multiple comparison method). The following figures are the same.

When cultured on PDA medium, the colony diameter of strain ML-2 increased significantly from 1.82 cm on day 3 to 7.60 cm on day 5. The relationship between colony diameter (Y, cm) and incubation time (X, days) was well described by the linear regression equation Y = 0.48X + 0.67 (R^2^ = 0.979, F = 143.88, *p* < 0.01). On SDAY medium, the colony diameter similarly increased from 1.96 cm to 7.41 cm over the same period, with a regression equation of Y = 0.45X + 0.84 (R^2^ = 0.987, F = 235.17, *p* < 0.01). Although colony diameters on PDA medium were consistently larger than those on SDAY throughout the 15-day cultivation period, the differences were not statistically significant (Figure 3).

**Figure 4:**
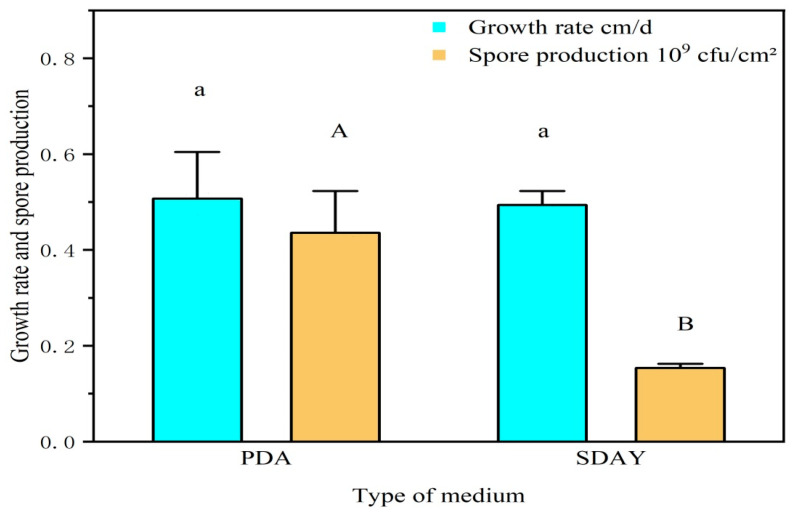
Growth rate and spore production of strain ML-2 on different media.

While strain ML-2 exhibited comparable growth rates on PDA medium (0.51 cm/d) and SDAY (0.49 cm/d) (*p* > 0.05), its sporulation was markedly influenced by medium composition. PDA medium supported significantly higher spore production (4.36 × 10^8^ spores/mL) than SDAY (1.54 × 10^8^ spores/mL), representing an approximately threefold increase (*p* < 0.01) (Figure 4).

### 3.3. Determination of Virulence of Strain ML-2 Against Different Life Stages of P. operculella

#### 3.3.1. Infection Symptoms at Different Life Stages of *P. operculella*

**Figure 5:**
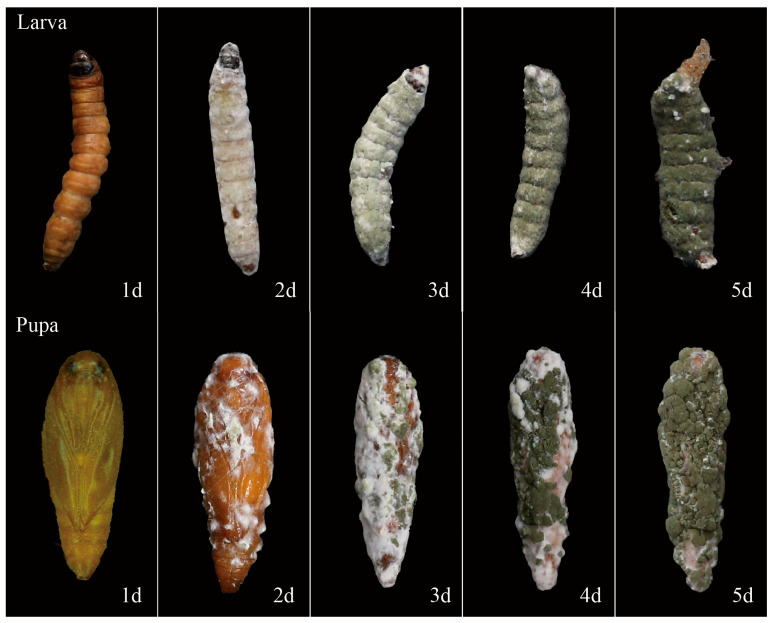
Infection symptoms of *M. robertsii* on larvae and pupae of *P. operculella*. (Larva) Diseased larva of *P. operculella*. (Pupa) Diseased pupa of *P. operculella*.

*P*. *operculella* larvae infected by strain ML-2 became stiffened and turned from white to yellowish-brown. Two days post-mortem, sparse white hyphae emerged across the larval surface. By the third day, profuse mycelial growth completely enveloped the cadaver, with scattered green conidia appearing on the white mycelium. After five days, the entire larval surface was densely covered by green conidia (Figure 5-Larvae). In infected pupae, the initial symptom was a color shift from yellow to yellowish-brown. White mycelia were first observed on second day post-infection. By day three, mycelia had proliferated extensively, encasing the pupal body and forming localized patches of green conidia. On the following day, abundant green conidia were produced on the pupal surface. Within five days, similar to infected larvae, the entire pupal cadaver was completely covered with green conidia (Figure 5-Pupae).

#### 3.3.2. Virulence Determination of Strain ML-2 Against Different Life Stages of *P. operculella*

**Figure 6:**
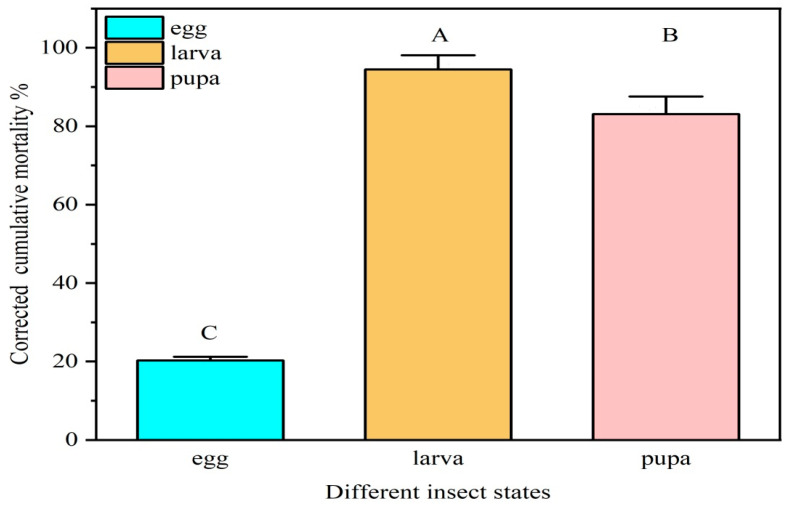
Corrected cumulative mortality of *M. robertsii* against different life stages on 7th Day. Larvae were significantly more susceptible than pupae and eggs (*p* < 0.01).

At a concentration of 1 × 10^8^ conidia/mL, strain ML-2 caused significantly different cumulative corrected mortality among the three developmental stages of *P. operculella* on seventh day post-treatment (F = 468.494, *p* < 0.01), with larvae being the most susceptible, followed by pupae and eggs (Figure 6). Corrected mortality rates reached 94.50% ± 1.77% in larvae and 83.07% ± 2.19% in pupae, whereas only 20.28% ± 0.39% of eggs were killed (Figure 6).

### 3.4. Time-Concentration-Mortality Model Analysis of Strain ML-2 Against P. operculella Larvae and Pupae

#### 3.4.1. Daily Cumulative Mortality of *P. operculella* Larvae and Pupae

**Figure 7:**
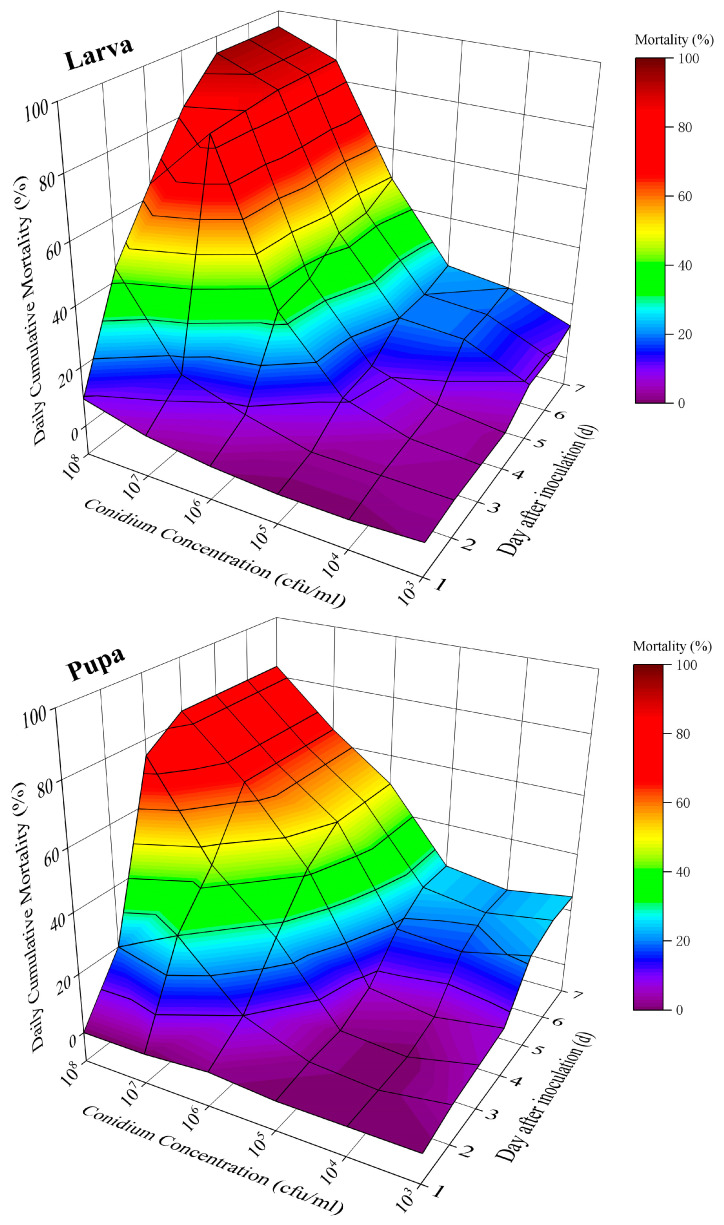
Daily cumulative mortality (mean ± SE) of 3-day-old larvae (Larva) and 1-day-old pupae (Pupa) following inoculation with serial concentrations (1 × 10^3^ to 1 × 10^8^ conidia/mL) of *M. robertsii* ML-2 over 7 days. Mortality increased with both time and conidial concentration.

For both larvae and pupae, cumulative mortality increased with both inoculation time and conidial concentration. From the fifth day post-inoculation onward, all spore suspension treatments resulted in significantly higher mortality than the control in both larvae (F = 694.71, *p* < 0.01) and pupae (F = 204.10, *p* < 0.01). On seventh day, larval mortality ranged from 12.01% to 94.50% across concentrations, while pupal mortality ranged from 21.67% to 83.07%. These results indicate that *P. operculella* larvae are more sensitive than pupae to variations in ML-2 conidial concentration (Figure 7).

#### 3.4.2. Infective Pathogenic Effects on *P. operculella* Larvae and Pupae

The observed responses of *P. operculella* mortality fit the TCM model (Table 1). Data for all the bioassays fit the TCM model with accepted homogeneity fit based on the Hosmer–Lemeshow statistic C (*p* ≥ 0.05); both models constructed for larvae and pupae were validated as TCM models, with the statistics for 3-day-old larvae (C = 16.54, df = 8, *p* = 0.056) and 1-day-old pupae (C = 14.04, df = 8, *p* = 0.081) passing the heterogeneity test. Furthermore, the t-statistics for all estimated dose effect and time effect parameters reached highly significant levels (*p* < 0.01).

In the TCM model, the dose effect parameters β for 3-day-old larvae and 1-day-old pupae were 0.748 and 0.538, respectively, indicating that *M. robertsii* exhibited strong pathogenic infectivity against both developmental stages of *P. operculella,* with larvae being more sensitive to changes in *M. robertsii* concentration. The ranking of the conditional time effect parameters (γj) for 3-day-old larvae was γ6 < γ7 < γ1 < γ2 < γ3 < γ4 < γ5, suggesting that the peak mortality period for larvae treated with *M. robertsii* occurred on day 5 post-inoculation. For 1-day-old pupae, the ranking of γj was γ7 < γ1 < γ6 < γ2 < γ4 < γ5 < γ3, indicating that the peak mortality period for pupae treated with *M. robertsii* occurred on day 3 post-inoculation. The estimated cumulative time effect (τj) at a given dose (di) increased with time post-inoculation, demonstrating that the cumulative mortality of larvae and pupae treated with the same concentration of *M. robertsii* increased over time.

#### 3.4.3. Dose Effect on *P. operculella* Larvae and Pupae

**Figure 8:**
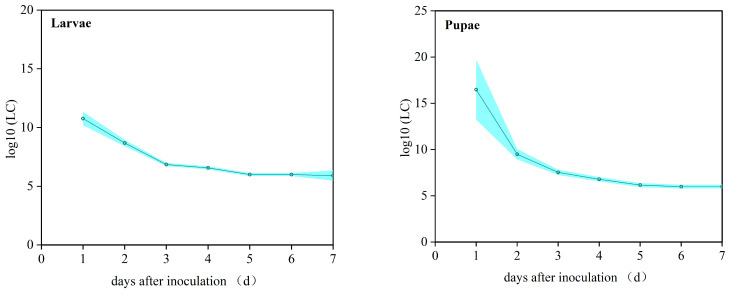
The logarithms value of the lethal dose of *M. robertsii* ML-2 against larvae and pupae of *P. operculella*. (Larvae) Logarithmic LC_50_ values for 3-day-old larvae. (Pupae) Logarithmic LC_50_ values for 1-day-old pupae.

TCM models were fitted using the DPS software system to generate predicted mortality responses of *P. operculella* following inoculation with *M. robertsii* strain ML-2 (Figure 6). For 3-day-old larvae, the LC_50_ values on days 3, 5, and 7 were 6.97 × 10^6^, 9.78 × 10^5^, and 8.03 × 10^5^ conidia/mL, respectively. For 1-day-old pupae, the LC_50_ values on the same days were 3.37 × 10^7^, 1.42 × 10^6^, and 9.64 × 10^5^ conidia/mL, respectively. These results indicate that *M. robertsii* strain ML-2 exhibits a clear dose-dependent effect on *P. operculella* larvae and pupae, and the LC_50_ values gradually decreased over time (Figure 8).

#### 3.4.4. Time Effect on *P. operculella* Larvae and Pupae

Based on TCM model predictions (Table 2), the LT_50_ values for 3-day-old *P. operculella* larvae inoculated with *M. robertsii* ML-2 at concentrations of 1 × 10^6^, 1 × 10^7^, and 1 × 10^8^ conidia/mL were 4.98, 2.89, and 2.34 days, respectively. For 1-day-old pupae, the corresponding LT_50_ values were 5.91, 3.70, and 2.72 days. At the highest concentration tested (1 × 10^8^ conidia/mL), the LT_90_ for larvae was 4.35 days, whereas the LT_90_ for pupae could not be estimated due to final mortality below 90%. These results demonstrate that, at equivalent concentrations, ML-2 exhibits a shorter lethal time against larvae than against pupae.

## 4. Discussion

Entomopathogenic microorganisms are key agents in the biological control of agricultural pests. To date, four groups of entomopathogens—bacteria, fungi, viruses, and nematodes—have been reported against *Phthorimaea operculella*. *Bacillus thuringiensis* is the most widely applied bacterium, yet its efficacy under field and storage conditions is debated [23]. Among fungi, *Metarhizium anisopliae*, *Metarhizium rileyi*, *Cordyceps tenuipes*, and *Beauveria bassiana* show control potential; *M. rileyi* is the most virulent against larvae but exhibits slow mycelial growth [24,25]. The *P. operculella* granulovirus has been widely used but faces resistance issues [26]. Nematodes such as *Steinernema carpocapsae* and *Heterorhabditis bacteriophora* are effective against prepupae but are environmentally demanding [27]. Given the limitations of current measures, there is an urgent need for novel entomopathogenic fungi. Unlike exotic isolates, *M. robertsii* strain ML-2 was isolated from naturally infected *P. operculella* cadavers in the field, suggesting native adaptation and ecological compatibility. This strain thus offers potential advantages in colonization, environmental resilience, and pathogenic stability.

The study revealed that the pathogenicity of *M. robertsii* against larvae and pupae was significantly stronger than that against eggs, which may be related to the inherent developmental processes of eggs and the hereditary protective traits conferred by parents [28]. For example, during embryonic development of *Tribolium confusum*, the serosa acts as an immune-active barrier, producing immune-related proteins and antimicrobial peptides to defend against microbial invasion [29]. Eggs of *Philanthus triangulum* release nitric oxide to kill soil molds, ensuring successful hatching in soil [30]. Furthermore, insect eggs harbor microbial communities that resist pathogens. For instance, bacterial communities on the surface of *Musca domestica* eggs effectively inhibit the growth of pathogenic fungi in animal feces [31]. Female *Megymenum gracilicorne* apply filamentous fungi to the egg surface, forming a dense physical barrier that significantly suppresses damage from pathogenic microorganisms and predators [32]. Additionally, both larvae and pupae of *P. operculella* possess numerous intersegmental membranes and spiracles, which facilitate the active attachment of conidia, thereby increasing the opportunities for *M. robertsii* to infect larvae and pupae.

This study employed the Time-Concentration-Mortality (TCM) model to analyze the virulence of *M. robertsii* strain ML-2 against *P. operculella* larvae and pupae. It was found that the estimated LC_50_ decreased over time, while the estimated LT_50_ decreased with increasing dose. Time and dose effects are key parameters for evaluating the insecticidal activity of biocontrol agents. Traditional probit analysis treats these two effects as independent variables and describes them with separate empirical formulas, making it difficult to reveal their interrelationship [33]. In contrast, the TCM model partially compensates for the limitation of treating time and dose effects as independent in probit analysis. It simultaneously analyzes the effects of time, dose, and their interactions within a single model, directly clarifying evaluation metrics such as lethal dynamics, peak mortality periods, and lethal doses, thereby more fully reflecting the completeness and objectivity of bioassay data.

Similar to other entomopathogenic fungi, *M. robertsii* infects insects by penetrating the host cuticle. A range of pathogenic mechanisms have been characterized in *Metarhizium* species, including the production of antifungal metabolites to suppress cuticular microorganisms, secretion of cuticle-degrading enzymes, and evasion of host immune responses [34,35,36,37]. Furthermore, some *Metarhizium* species can also establish rhizosphere competence, switching from a plant-associated lifestyle to an insect-pathogenic lifestyle when a host is present [38,39], and possess sophisticated nutrient-sensing pathways to ensure survival under stress [40]. In this study, the locally isolated *M. robertsii* strain ML-2 exhibited a rapid lethal effect on *P. operculella* larvae, with an LT_50_ value of 2.34 days. This value is comparable to that reported for *Beauveria bassiana* (2.42 days) and lower than those reported for *M. anisopliae* (4.85 days) and *Isaria tenuipes* (4.8 days) against the same pest [22,25,41]. Considering its native origin and high virulence under laboratory conditions, *M. robertsii* strain ML-2 represents a promising candidate for further development as a biocontrol agent against *P. operculella*, pending field validation.

The type of culture medium greatly influences the sporulation yield, growth rate, and insecticidal activity of biocontrol fungi. For instance, PPDA medium (PDA medium + 5% peptone) is conducive to sporulation of *Metarhizium* sp. pathogenic to *Plutella xylostella*, Starch Agar medium promotes mycelial growth of the biocontrol fungus *Penicillium ochrochloron*, and PDA medium enhances sporulation of *P. ochrochloron* [42]. This study found that *M. robertsii* strain ML-2 grew rapidly on both PDA medium and SDAY media, with no significant difference in colony growth rates between the two media. However, when sporulation yield was measured on the 15th day, the PDA medium produced approximately three times more spores than the SDAY medium, indicating that PDA medium is more conducive to sporulation of *M. robertsii* strain ML-2. This may be attributed to differences in carbon-to-nitrogen ratio and nitrogen sources. The nitrogen source in PDA medium is derived from potato starch, which releases nitrogen relatively slowly, whereas the nitrogen sources in SDAY medium, primarily peptone and yeast extract powder, release nitrogen more rapidly. The mechanisms of carbon and nitrogen metabolism in pathogenic fungi are known to directly influence their growth, reproduction, and pathogenicity [43]. It has been reported that a lower carbon-to-nitrogen ratio facilitates conidial formation in biocontrol fungi, whereas a higher ratio promotes mycelial growth. Moreover, the optimal types of carbon and nitrogen sources for mycelial growth and spore formation also differ [44]. The findings of this study are limited to growth rates and sporulation yield under laboratory conditions, and the adaptability of *M. robertsii* strain ML-2 to natural field environments requires further investigation.

## 5. Conclusions

*Metarhizium robertsii* is an entomopathogenic fungus that can infect the potato tuber moth, *Phthorimaea operculella*. In the present study, *M. robertsii* strain ML-2 demonstrated high virulence against both larvae and pupae of *P. operculella* under laboratory conditions and can be readily cultured on PDA medium. These results highlight its significant potential as a biocontrol agent for this pest, pending further field validation.

## Figures and Tables

**Table 1:** TCM model simulation and parameter estimation of larvae and pupa treated with *M. robertsii* ML-2.

Insect State	Conditional Mortality Model				Cumulative Mortality Model			
	Parameter ^a^	Value	SE	T ^b^	Parameter ^a^	Value	Var (τj)	Cov (τj, βj)
3-day-old larva	β	0.748	0.046	16.438	β	0.748	0.004	0.004
	γ 1	−8.412	0.440	19.138	τ1	−8.412	0.329	−0.026
	γ 2	−7.084	0.364	19.446	τ2	−6.849	0.216	−0.025
	γ 3	−5.778	0.328	17.621	τ3	−5.483	0.181	−0.024
	γ 4	−6.915	0.389	17.797	τ4	−5.269	0.179	−0.024
	γ 5	−5.908	0.314	18.836	τ5	−4.845	0.161	−0.023
	γ 6	−18.800	0.000	18 × 10^8^	τ6	−4.845	0.161	−0.023
	γ 7	−7.562	1.887	4.007	τ7	−4.781	0.165	−0.015
1-day-old pupa	β	0.538	0.060	8.984	β	0.538	0.007	0.007
	γ 1	−9.236	1.206	7.659	τ1	−9.236	2.731	−0.044
	γ 2	−5.500	0.443	12.411	τ2	−5.477	0.367	−0.046
	γ 3	−4.846	0.432	11.208	τ3	−4.419	0.337	−0.046
	γ 4	−5.133	0.422	12.161	τ4	−4.020	0.315	−0.045
	γ 5	−4.919	0.374	13.144	τ5	−3.679	0.279	−0.042
	γ 6	−6.033	0.481	12.551	τ6	−3.588	0.272	−0.042
	γ 7	−16.290	0.000	16.3 × 10^8^	τ7	−3.588	0.272	−0.042

^a^ The subscript number represents the specific day after inoculation. ^b^ The t statistics were highly significant for all the parameters estimated (*p* < 0.0001).

**Table 2:** TCM model-based estimation of LT_50_ and LT_90_ for larvae and pupae infected by *M. robertsii* ML-2.

Insect State	LT_50_/LT_90_ (d)	Lethal Time at Different Concentrations/d		
		1 × 10^6^	1 × 10^7^	1 × 10^8^
3-day-old larva	LT_50_	4.98	2.89	2.34
	LT_90_	—	—	4.35
1-day-old pupa	LT_50_	5.91	3.70	2.72
	LT_90_	—	—	—

## Data Availability

The original contributions presented in this study are included in the Article/Supplementary Material. Further inquiries can be directed to the corresponding authors.

## Supplementary Materials

The following supporting information can be downloaded at: https://www.mdpi.com/article/10.3390/insects17050474/s1, Table S1: The primers used in this study; Table S2: Type or reference strains of *Metarhizium robertsii* and related PARB species used in this study; Table S3: Results of sequence alignment analysis between strain ML-2 and the type strain sequences.
